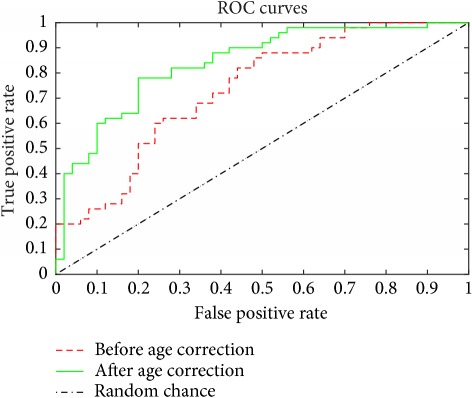# Study of the Influence of Age in ^18^F-FDG PET Images Using a Data-Driven Approach and Its Evaluation in Alzheimer's Disease

**DOI:** 10.1155/2018/3786083

**Published:** 2018-02-08

**Authors:** Jiehui Jiang, Yiwu Sun, Hucheng Zhou, Shaoping Li, Zhemin Huang, Ping Wu, Kuangyu Shi, Chuantao Zuo, Alzheimer's Disease Neuroimaging Initiative

**Affiliations:** ^1^Shanghai Institute for Advanced Communication and Data Science, Shanghai University, Shanghai, China; ^2^PET Center, Huashan Hospital, Fudan University, Shanghai, China; ^3^Department of Nuclear Medicine, Technische Universität München, Munich, Germany; ^4^Institute of Functional and Molecular Medical Imaging, Fudan University, Shanghai, China

## Abstract

**Objectives:**

^18^F-FDG PET scan is one of the most frequently used neural imaging scans. However, the influence of age has proven to be the greatest interfering factor for many clinical dementia diagnoses when analyzing ^18^F-FDG PET images, since radiologists encounter difficulties when deciding whether the abnormalities in specific regions correlate with normal aging, disease, or both. In the present paper, the authors aimed to define specific brain regions and determine an age-correction mathematical model.

**Methods:**

A data-driven approach was used based on 255 healthy subjects.

**Results:**

The inferior frontal gyrus, the left medial part and the left medial orbital part of superior frontal gyrus, the right insula, the left anterior cingulate, the left median cingulate, and paracingulate gyri, and bilateral superior temporal gyri were found to have a strong negative correlation with age. For evaluation, an age-correction model was applied to 262 healthy subjects and 50 AD subjects selected from the ADNI database, and partial correlations between SUVR mean and three clinical results were carried out before and after age correction.

**Conclusion:**

All correlation coefficients were significantly improved after the age correction. The proposed model was effective in the age correction of both healthy and AD subjects.

## 1. Introduction

The influence of age has been proven to be the greatest interfering factor for many clinical dementia diagnoses [1]. For Alzheimer's disease (AD), the most common (60%–70%) form of chronic neurodegenerative dementia in the elderly [2], advanced age is commonly associated with adverse changes in the brain that are reflected in different imaging modalities such as magnetic resonance imaging (MRI), functional MRI (fMRI), and positron emission tomography (PET) [3–6]. Thus, it is important for clinics to eliminate the influence of age in order to determine whether the abnormalities in specific regions correlate with normal aging, disease or both. For instance, several research studies have reported that the grey matter volume and cortical thickness within certain regions-of-interest (ROIs) such as the hippocampus, the inferior frontal, superior frontal, middle frontal, and temporal cortices decreased with aging, based on the findings of structural magnetic resonance imaging (MRI) [7–10]. The amyloid *β* level, as measured by the standardized uptake value ratio (SUVR) in ^11^C-Pittsburgh Compound B (PiB) PET scans was found to increase abnormally after the age of 70 in apolipoprotein E (APOE) *ε*4 Genotype carriers [11]. The important parameter of fractional anisotropy (FA, a measure of the degree of anisotropy of a diffusion process) was found to exhibit a negative linear correlation with age in most white matter regions, in particular in the superior corona radiata [7].

Currently, ^18^F-Fluorodeoxyglucose (FDG) PET is regarded as a “Gold Standard” for AD diagnosis, since its predictive value enables detection of abnormal cerebral metabolism during the earliest stages of dementia or even before the dementia is present [4, 12–14]. It is a major challenge for radiologists to eliminate the influence of age from ^18^F-FDG PET images. For this purpose, a few research groups have investigated the manner in which age-influenced brain metabolism occurs and within which brain regions. For example, strong negative correlations were found between metabolism and normal aging mainly in the frontal and temporal lobes [15–22], suggesting that tissue shrinkage or loss and glucose metabolism reduction could be accelerated by normal aging. In other studies [23–25], regions within a default mode network (DMN) including the temporal, parietal, and prefrontal areas and the posterior cingulate cortex were found to be significantly reduced with age. In view of these findings, it is reasonable to suggest that image intensity of certain regions, especially the prefrontal and temporal areas, may exhibit double the effect of normal aging.

More specifically, in [26], the authors applied an age-correction criterion [27] based on the results of the Montreal Cognitive Assessment (MoCA) according to the circumstance that predetermined the age range of the patient. Similar criteria can be found in [28]. Although these corrections were not based directly on brain images, the line of thought was illuminating. Subsequently, [29] using MRI, a simple linear model was proposed to correct the effect of age on grey matter (GM) voxel values by determining a regression coefficient *β*_*C*_ between age and each voxel coordinate of the separate healthy control group. This *β*_*C*_ value indicated the declination speed of the corresponding voxel with increasing age. The same model was used for the MRI GM voxel values and the ^18^F-FDG PET intensity values by [14]. Moreover, as proposed in previous studies, it was unnecessary to apply the linear correction model to all the voxels within the images, because not all regions correlated significantly with age [17, 19–21]. However, there have been a lack of studies investigating the exact brain regions in ^18^F-FDG PET images that have negative relationships with age, and these have defined any age-correction mathematical models.

Therefore, the present study aimed to (1) use a data-driven method to explore brain regions that have a strong negative linear relationship with age in ^18^F-FDG PET images and to then define an age-correction mathematical model. Subsequently, the intent was to (2) validate those brain regions and the mathematical modal using statistical analysis on both healthy and AD subjects.

## 2. Materials and Methods

### 2.1. Datasets

The two datasets used in this study were as follows: (1) 255 healthy control ^18^F-FDG PET images were from Huashan Hospital, Fudan University, Shanghai, China; (2) 262 healthy control and 50 AD ^18^F-FDG PET images were selected from Alzheimer's Disease Neuroimaging Initiative (ADNI).

For the first dataset, data collection was approved by the Medical Ethics Committee of Huashan Hospital, Fudan University, Shanghai, China. All subjects signed agreements to participate in this study. The sample group was comprised of 127 female and 128 male healthy subjects (ages: 20–79), who had no current axis *I* psychiatric disorder, no psychotropic medication use or hormone use within the prior 6 months, and no history of head injury or alcohol abuse. These subjects received brain ^18^F-FDG PET scans. No occult ^18^F-FDG-avid carcinoma was determined in any of the included subjects, based on the brain PET examination. Blood glucose levels were monitored prior to ^18^F-FDG injection (5.5 ± 0.8 mm for males and 5.3 ± 0.9 mm for females).

The 131 male and 131 female healthy subjects (age: 56.5–80) were selected from the ADNI database. Furthermore, the 25 male and 25 female AD subjects (ages: 59.3–79.8) were also selected from the ADNI database.  Table 1 shows the basic information for the datasets.

### 2.2. Image Acquisition

All subjects who underwent ^18^F-FDG PET brain scans at Huashan Hospital were in a resting state. The ^18^F-FDG PET whole-body scans followed the brain scans in case of these subjects (1.5 min/bed, 5 beds). A 222–296 MBq injection of ^18^F-FDG was administered intravenously under standardized conditions (in a quiet, dimly lit room with the patient's eyes open). A 10 min 3-dimensional brain emission scan was acquired at 45 min after injection with a state-of-the-art PET scanner (Siemens Biograph 64 HD PET/CT, Siemens, Germany). During the scanning procedure, the subjects' heads were immobilized using a head holder. Attenuation correction was performed using a low-dose CT (150 mAs, 120 kV, Acq. 64 × 0.6 mm) prior to the emission scan. Following corrections for scatter, dead time, and random coincidences, PET images were reconstructed by 3-dimensional filtered backprojection and a Gaussian Filter (FWHM 3.5 mm), providing 64 contiguous transaxial slices of 5 mm thick spacing.

For images downloaded from the ADNI database, detailed information regarding the data acquisition protocol is publicly available on the LONI website (https://ida.loni.usc.edu/login.jsp). Briefly, PET images from the ADNI database were acquired from a variety of scanners nationwide using either a 30-minute six frame scan or a static 30-minute single frame scan, both acquired 30–60 minutes after injection. For the former case, the dynamic scans were coregistered with the first frame and averaged to create a single average image.

### 2.3. Image Preprocessing

The aim of preprocessing was to remove unwanted distortions such as low-frequency background noise, to spatially normalize the images into a standard space defined by template images and to enhance important image features prior to further computational processing. In the present study, all of the images were preprocessed using Statistical Parametric Mapping 8 (SPM8), a Statistical Parametric Mapping software package designed for the analysis of brain imaging data sequences (http://www.fil.ion.ucl.ac.uk/spm/software/spm8/), implemented in MATLAB R2014a. The preprocessing was mainly composed of normalization and smoothing. The details of image preprocessing can be found in the following paragraph.

All original DICOM images from each subject were combined into one NIfTI format file using a tool in MRIcron called DCM2NII (available at http://people.cas.sc.edu/rorden/mricron/index.html). For each subject, the PET image was first normalized to the Montreal Neurological Institute (MNI, McGill University, Montreal, Canada) space through the “Normalize: Estimate and Write” methodology. During this step, a reference PET template provided by SPM software was used as the standard space. The nonlinear warping normalization was carried out automatically following determination of the optimum 12-parameter affine transformation. Subsequently, the normalized images prefixed with a* “w”* were smoothed using an isotropic Gaussian smoothing kernel with the full-width at half maximum (FWHM) of 10 × 10 × 10 mm^3^. Thus, the images' noise-signal ratio can be improved by these standardized processing steps. The resulting images had 91 × 109 × 91 voxels with a voxel size of 2 × 2 × 2 mm^3^. Next, the preprocessed FDG PET 3D images were concatenated to one 4D image file using the DCM2NII tool in preparation for the following data analysis.

### 2.4. Exploration of Brain Regions Displaying Negative Relationships with Age

To explore brain regions with negative relationships with age, three steps were used in this section: (1) A data-driven method, Functional MRI of the Brain's (FMRIB's) Linked Independent Component Analysis (FLICA), was used to obtain an indication of the initial brain regions that had a strong negative relationship with age. (2) To obtain a more accurate indication of age-related brain regions, different thresholds of voxel values were set and compared correlations between SUVR mean and age using Pearson correlation. (3) Optimized brain regions were defined after the application of suitable threshold voxel values.

#### 2.4.1. Exploration of the Initial Age-Related Brain Regions Using FLICA

To define initial brain regions, a data-driven approach—FLICA—was used. Independent component analysis (ICA) is a computational model for separating a multivariate signal into additive subcomponents. Applied in the spatial dimension, it is efficient for finding meaningful and spatially independent components by assuming that the subcomponents are non-Gaussian distributed spatial sources and thus they are likely to represent real underlying structured features in the dataset. This is because, during linear mixing processes, it is likely that the non-Gaussian independent sources can turn into more Gaussian observed signals. Consequently, seeking non-Gaussian sources can be an unsupervised means of revealing the original independent sources.

FLICA is an advanced independent component analysis (ICA) approach implemented in FSL running as a MATLAB toolbox in the Linux system [30, 31]. FLICA is an entirely data-driven approach that can comodel multiple imaging modalities. Its main goal is to model the imaging data as a set of interpretable features (independent components), most of which characterize biophysically plausible modes of variability across all of the subjects' images. Unlike in a principal component analysis, the mixing matrix vectors of an ICA are not forced to be orthogonal to each other and thus can explain common variance of variables external to the ICA, such as age [32]. Linked ICA has also been implemented in age studies in earlier papers [32, 33]. (https://fsl.fmrib.ox.ac.uk/fsl/fslwiki/FLICA).

In the present study, the dataset from Huashan Hospital was analyzed using FLICA, which was run totally on MATLAB R2014a installed in a Linux environment. The comprehensive library of analysis tools for brain imaging data, FSL, was also required for the procedure. The combined 4D image file was converted to a readable format in FSL (*∗*.nii.gz) by the DCM2NII tool. Following data preparation and creation of the environmental variables and options, the analysis was run with all automatic calculations. When finished, the output was saved safely, including a set of independent components, in a sensible order (either in the order of total energy or to match with a previous similar run), with the relevant spatial maps.

**Figure 1:**
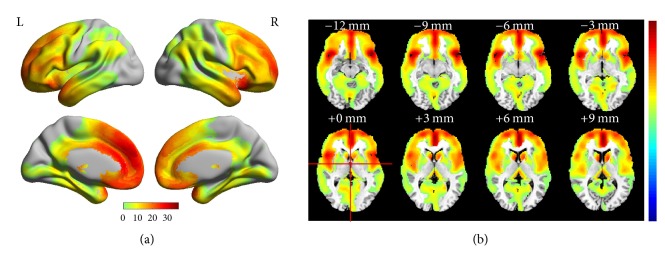
Anatomic structure of the brain regions corresponding to the significant age-related IC3 (pseudocolor) obtained across all 255 healthy subjects.

The independent components (ICs) output from FLICA contained three types of information: IC values, spatial information, and voxel values (see Table 2). The IC values were represented as an *M* × *N* matrix where *M* is the number of the subjects and *N* is the number of ICs obtained. The spatial information (SI) and corresponding voxel values (VV) of the ICs were combined in a 4D nii formatted file that could be opened and viewed as a common brain image where voxel values can be displayed in pseudocolor (Figure 1). Note that the voxel value is the probability operation result of the coordinate of each voxel. That is, the possibility of the region having a strong relationship with age becomes larger as the voxel values of the corresponding coordinates become larger. Hence, an appropriate threshold of VV, *σ*, is required to be defined in order to obtain an accurate indication of affected brain regions.

#### 2.4.2. Further Exploration of the Final Age-Related Brain Regions by Defining *σ*

Three steps were used to achieve appropriate values of *σ*:

(I) Extraction of initial brain regions: for 255 image data from Huashan Hospital, SI corresponding to the IC was extracted as initial brain regions using the Automatic Anatomical Labelling (AAL) template in the Montreal Neurological Institute (MNI) standard space.
(II) Definition of *σ*: the initial brain regions were then optimized by setting different thresholds for *σ*. In particular, *σ* was set to start as 1 and end as the maximal voxel value, *X*. To generate an optimized brain region mask for each *σ* separately, all voxels whose values were no less than *σ* were set as 1 and others as 0. Next, data from the 255 healthy subjects were utilized to calculate SUVR mean [34] of the targeted brain regions generated with different *σ*, by using the paracentral lobule (AAL 69 and AAL 70) as the reference regions [35]. The formula to form SUVR mean is [34]
  (1) $$\begin{array}{c} SUVR\;\;mean=\frac{I_{avg\_ROIC}}{I_{avg\_ref}}, \end{array}$$
  where *I*_avg_ROIC_ is the average intensity of the brain regions and *I*_avg_ref_ is the average intensity of the reference region.
(III) Comparison experiments: subsequently, Pearson correlation [36] was used to analyze correlations between age and SUVR mean under different *σ* and the most appropriate *σ* was selected to achieve the strongest correlations. In order to reduce the influence of accidental factors to a minimum, 250 subjects were selected randomly from a total of 255 subjects for 3,000 times and the Pearson correlation analysis was carried out separately on these data. The statistical results, including the correlation coefficients *R* and *p* values, were taken as an average of the 3,000 results.

### 2.5. Definition of the Age-Correction Mathematical Model

With an appropriate *σ*, Pearson correlation analysis was carried out again on the same 255 healthy subjects in order to obtain the linear coefficient *β*_*C*_. The entire process was as follows:

(I) The linear regression coefficient *β*_*C*_ between age and the corresponding set of SUVR means was calculated. Thus, the development of SUVR mean during normal aging can be described as
  (2) $$\begin{array}{c} SUVR\;\;mean=\beta_{C}\times age+p_{2}. \end{array}$$
(II) Similar to Section 2.4.1, the linear regression process was repeated for 3,000 times and the *β*_*C*_ value was taken as an average of the 3,000 results.
(III) Finally, to carry out the age correction, a model similar to the one proposed in [29] was defined, and the amount (Δ) assumed to have been affected by the subject's age since birth (i.e., aged 0) was calculated:
  (3) $$\begin{array}{c} \Delta =Y\left(\;{age}_{subject}\;\right)-Y\left(\;{age}_{\text{0}}\;\right)=\beta_{C}\times age. \end{array}$$

Then the target index was then corrected by subtracting this amount, as follows:

(4) $$\begin{array}{c} {Index}_{cor}={Index}_{tar}-\Delta . \end{array}$$

### 2.6. Evaluation of the Brain Regions and Age-Correction Mathematical Model

To validate the effectiveness of the brain regions and age-correction mathematical model defined in the present study, they were evaluated using another dataset that included 262 healthy subjects and 50 AD subjects from the ADNI. These two datasets were firstly preprocessed using the same approach as in Section 2.3. Following preprocessing, the evaluation was composed of four tests.

- In the first test, using 262 healthy subjects, the correlations were calculated between age and SUVR mean in the final brain regions from Section 2.4.2. It was verified whether negative relationships between age and SUVR means also occurred in the new dataset. Pearson correlation analysis was used in this test.
- In the second test, 262 healthy subjects were used to test the effectiveness of the age-correction mathematical model. Pearson correlation analysis was also used to compare negative relationships between age and SUVR means before and after correction. As expected, the relationships should be weaker after correlation and the slope should be flat.
- In the third test, to visualize the changes before and after corrections, a case study with one healthy and one AD subject was evaluated.
- In the fourth test, to verify whether the age-correction mathematical model can be used for clinical purposes, 50 AD subjects from ADNI were tested. Partial correlation analyses were made between SUVR means and the outcomes of the two clinical scales, MMSE and CDRSB, and an imaging index, FDG (Table 1), and the results of the analyses were compared. During the partial analyses, the effects of education, sex, and ApoE4 status were removed as a set of controlled variables.
- In the final test, to verify whether the diagnostic accuracy of AD increased after the age correction in these 50 subjects, we performed principal component analysis (PCA) to extract image features and support vector machine (SVM) for AD classification. For simplification, linear kernel was used for SVM. 50 age-matched healthy subjects were selected from the 262 healthy subjects, and classification was carried out on these altogether 100 subjects before and after the age correction, respectively. The results are represented as the classification accuracy and ROC curves.

## 3. Results

### 3.1. Exploration of Brain Regions Displaying Negative Relationships with Age

#### 3.1.1. Exploration of the Initial Age-Related Brain Regions Using FLICA

**Figure 2:**
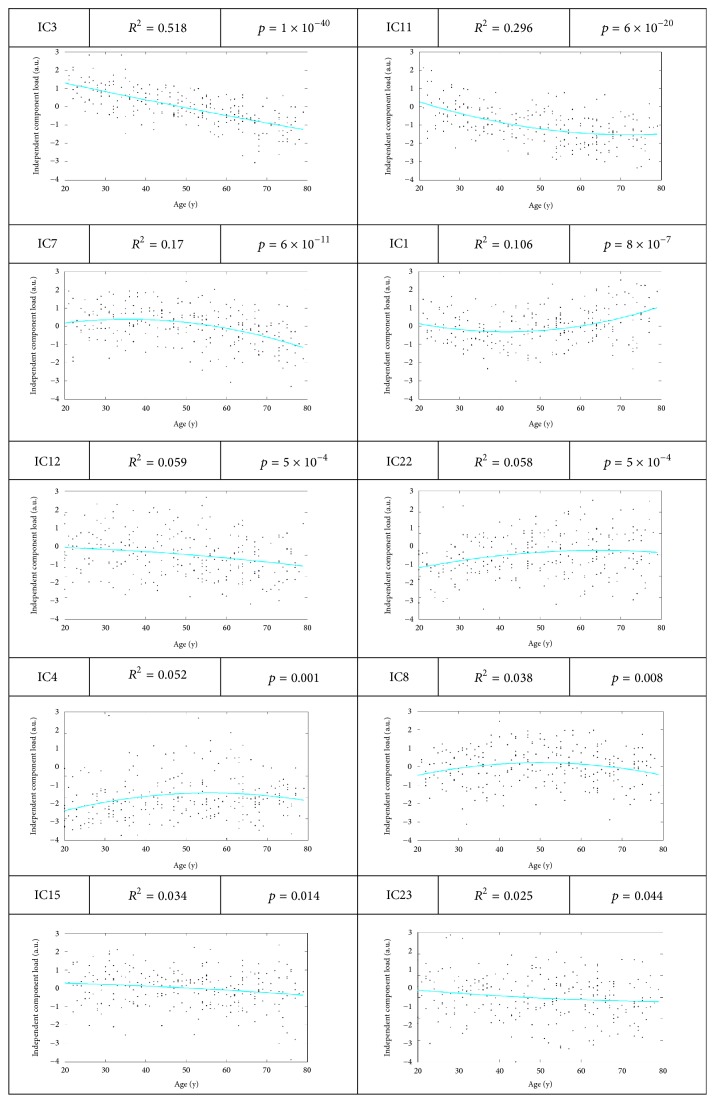
Plots of the post hoc relationship between the ICs and age.

A total of 29 ICs were obtained from the FLICA blind tests for any other demographics or cognitive factors of the participants. According to the purpose of the present study, there were 10 statistically significant components (*p* < 0.05), as shown in Figure 2. Amongst these, only the third component (IC3) revealed a monotone linear decrease with increasing age (although assessed using a quadratic fit) with a clear practical significance (*R*^2^ = 0.518, *p* = 1 × 10^−40^). *R* squared values for all other components were below 0.3. In the present study, both linear and quadratic fits were used to assess the relationship between IC values and age. As a result, the statistics of the quadratic fits were generally better than those of the linear fits. Furthermore, the curves of IC3 plotted with a linear fit and a quadratic fit, respectively, virtually coincide with each other. Thus, only results of quadratic fits are presented here. Moreover, the statistical results (quadratic fits) between the ICs and age in the 6 age groups of the 255 subjects are shown in Table 3. Amongst these, the subgroups of age 51–60 and 61–70 reached statistical significance (*p* < 0.05), while from the perspective of cumulation, the correlation reached statistical significance from the second age group 20–40.

Spatially, IC3 covered the regions encompassing: Frontal_Mid, Frontal_Med_Orb, Cingulum_Ant, Cingulum_Post, Parietal_Inf, Temporal_Mid, Temporal_Inf, Frontal_Sup, Frontal_Sup_Orb, Frontal_Mid_Orb, Frontal_Inf_Oper, Frontal_Inf_Tri, Frontal_Inf_Orb, Insula, Cingulum_Ant, Hippocampus, ParaHippocampal, Lingual, Fusiform, Caudate, Temporal_Sup, Temporal_Pole_Sup, and Temporal_Mid (all coordinating with the AAL template within the MNI standard space). The anatomic structure of these regions is shown in Figure 1.

The anatomic structure of the brain regions corresponding to the significant age-related IC3 degradation is illustrated in Figure 1. The voxel values ranged from 1 to 38. The total voxel volume was 128271.  Figure 1(a) was visualized using the BrainNet Viewer package (https://www.nitrc.org/projects/bnv/).  Figure 1(b) was visualized using the RESTplus toolkit (http://restfmri.net/forum/index.php?q=rest).

#### 3.1.2. Further Exploration of the Final Age-Related Brain Regions by Defining *σ*

**Figure 3:**
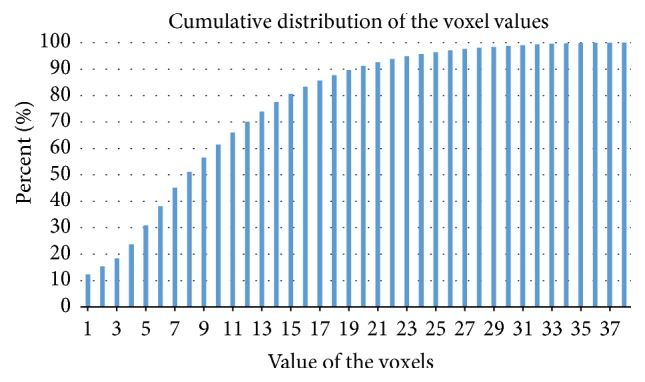
Cumulative distribution of the voxel values.

The automatic results from FLICA showed that IC3 totally contained 128271 voxels, with voxel values ranging from 1 to 38. The cumulative distribution of the 38 voxel values is shown in Figure 3. It can be observed that voxels with values of 1 to 9 occupied more than 50% of the IC3 region. As *σ* increased, the brain regions narrowed and the high voxel values were mainly concentrated in the Frontal_Inf_Orb, Insula_L, and Temporal_Pole_Sup_L. When *σ* is 38, the volume of the narrowed brain regions was only 16.

The statistical results of the Pearson correlation analyses between SUVR mean and age under different *σ*  (average of 3,000 times) are shown in Table 4. The correlation coefficients *R* became larger when *σ* increased and peaked at *σ* = 30 (*R* = −0.787; *p* value = 1.77*E* − 53). The fluctuation of *R* values can be observed when *σ* is greater than 30. According to previous literature, this may be due to the fact that the SUVR mean value of the IC3 region becomes vulnerable when *σ* is above the peak [34]. Note that all of these were negative, indicating a negative correlation between SUVR mean and age. In addition, all *p* values were less than 1.00*E* − 35, suggesting that the correlations were strong and statistically significant for every *σ* from 1 to 38.

**Figure 4:**
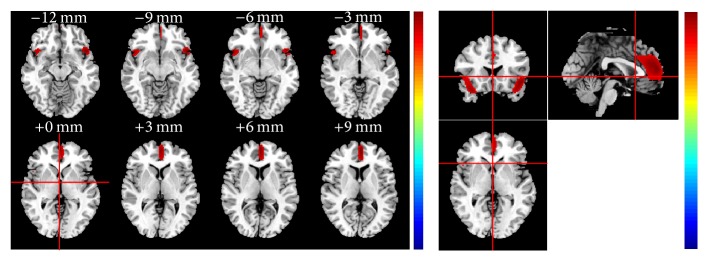
Anatomic structure of the final brain region with *σ* = 30.

According to the analysis results listed in Table 4, 30 was chosen as the best *σ* due to the strongest correlation. The final brain region was then defined. The number of voxels with *σ* = 30 was 2031. The AAL regions covered by the final brain region are shown in Table 5. The anatomic structure of the final brain region with *σ* = 30 is shown in Figure 4. In particular, areas corresponding to *σ* = 30 overlapped with 9 regions in AAL: Frontal_Inf_Orb_L (15), Frontal_Inf_Orb_R (16), Frontal_Sup_Medial_L (23), Frontal_Med_Orb_L (25), Insula_R (30), Cingulum_Ant_L (31), Cingulum_Mid_L (33), Temporal_Pole_Sup_L (83), and Temporal_Pole_Sup_R (84).

The final brain region included the bilateral orbital part of inferior frontal gyrus (AAL 15 and AAL 16), the left medial part of superior frontal gyrus (AAL 23), the left medial orbital part of superior frontal gyrus (AAL 25), the right insula (AAL 30), the left anterior cingulate (AAL 31), the left median cingulate, and paracingulate gyri (AAL 33), and bilateral superior temporal gyri (AAL 83 and AAL 84).

### 3.2. Definition of the Age-Correction Mathematical Model

The linearity coefficients *β*_*C*_ were calculated to create the corresponding age-correction mathematical model. Similar to the last step, random sampling was used in this step. 3,000 times of random sampling of 250 subjects from 255 healthy subjects were conducted and the linear regression coefficients *β*_*C*_ between their SUVR mean and age were calculated, respectively (the third column in Table 5). Thus, the mathematical model can be described as

(5) $$\begin{array}{c} SUVR\;\;mean=-0.00415\times age+p_{2}, \end{array}$$

where *β*_*C*_ = −0.00539 and *p*_2_ was the constant term when conducting the linear regression, which had no effect on the definition of the age-correction model.

### 3.3. Evaluation of the Mathematical Age-Correction Model in Healthy Participants

#### 3.3.1. Evaluation of the Final Brain Region in 262 Healthy Participants

**Figure 5:**
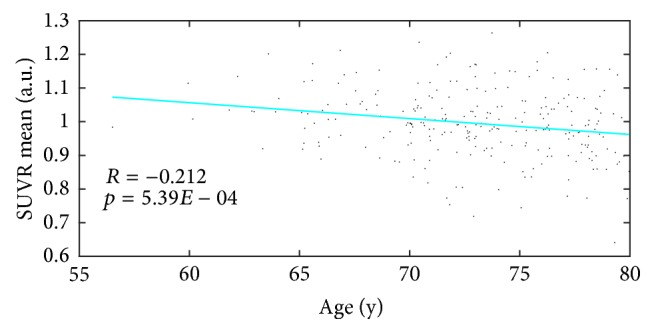
Scatter plot and fitting curves between SUVR mean formed from brain regions and age.

The validation results of the final brain region with the new dataset are shown in Figure 5. The *p* value was 5.39*E* − 04, indicating a statistically significant relationship between SUVR mean and age, and the correlation coefficient *R* = −0.212, indicating a negative correlation (see Figure 5).

#### 3.3.2. Evaluation of the Mathematical Age-Correction Model in 262 Healthy Participants

**Figure 6:**
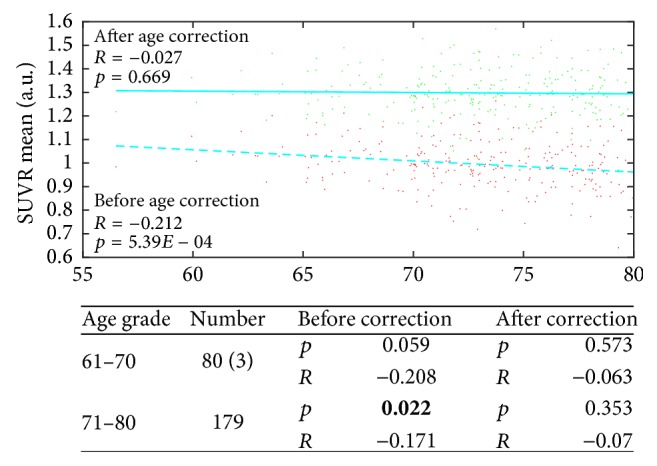
Scatter plot and fitting curves between SUVR mean and age across the 262 healthy subjects before (red and dashed) and after (green and solid) age correction. In addition, the statistical results of the age correction in the two age groups of the 252 healthy subjects are presented below.

The validation results are shown in Figure 6 as a scatter plot and fitting curves between SUVR mean and age across the 262 healthy subjects before (red and dashed) and after (green and solid) age correction. The correlation between SUVR mean and age measured using Pearson correlation analysis became weaker after age correction (the Pearson correlation coefficient *R* declined from 0.212 to 0.027 while the *p* value rose from 5.39*E* − 04 to 0.669), indicating that the influence of age on the regions used to form the SUVR mean was weakened as a result of age correction. Simultaneously, after correction, the slope was decreased to almost 0, indicating that the influence of age on the SUVR mean values had been eliminated. In addition, similar changes were found in the *p* values and Pearson correlation coefficients *R* in both age groups of the 252 healthy subjects after age correction (the Pearson correlation coefficients *R* declined from 0.208 to 0.063 for age group 61–70 and from 0.171 to 0.07 for age group 71–80 while the *p* value rose from 0.059 to 0.573 for age group 61–70 and from 0.022 to 0.353 for age group 71–80).

#### 3.3.3. A Qualitative Case Study

**Figure 7:**
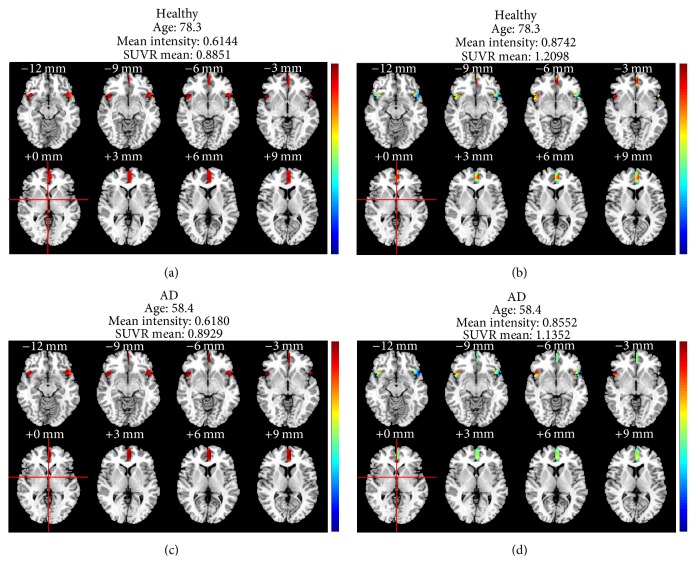
Visual result demonstration of a qualitative case study.

To visually demonstrate the correction results visually, one typical subject was selected from each of the two groups (healthy group and AD group) and the age-correction model was applied on all the voxels within the final brain region (the subject selected from the healthy group was aged 78.3 and the other subject selected from the AD group was aged 58.4). As shown in Figures 7(a) and 7(c) the origin anatomic structures of the final brain region are extracted from the healthy and AD subject, respectively. The corrected anatomic structures of the final brain regions extracted from the healthy and AD subject are shown in Figures 7(b) and 7(d), respectively. The mean intensities of these two subjects were very similar (0.6180 and 0.6144, resp.). The age correction results of the two subjects are shown in Figures 7(b) and 7(d), and the mean intensity of the healthy subject went beyond that of the AD subject (0.8742 and 0.8552, resp.), indicating a healthier status after eliminating the influence of age.

Additionally, the SUVR mean values based on the final brain region of each subjects in both the healthy and AD groups were calculated before and after the age correction and the statistical significance between the two groups was measured by an independent *t*-test. The *p* value declined from 1.706*E* − 08 to 1.740*E* − 10 after the age correction, indicating that the difference between healthy and AD subjects became more significant after the age correction.

#### 3.3.4. Evaluation of the Mathematical Age-Correction Model in AD Participants

**Figure 8:**
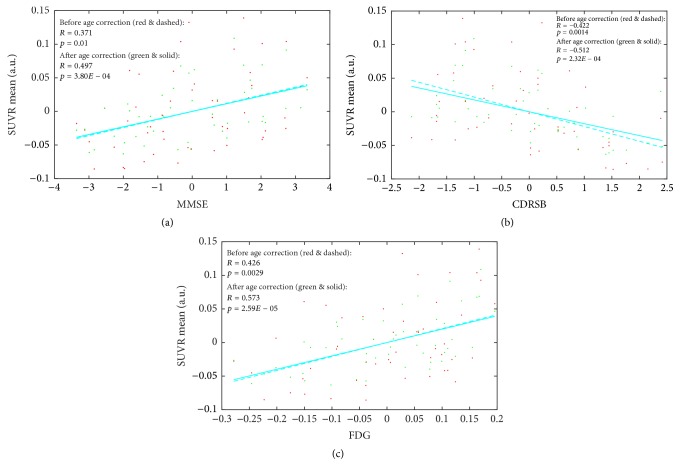
Scatter plot and fitting curves between SUVR mean and (a) MMSE, (b) CDRSB, and (c) FDG across the 50 AD subjects before (red dots and dashed lines) and after (green dots and solid lines) age correction.

To validate whether the age-correction model can serve to eliminate the confusing factor of age so as to highlight the pathological differences, the model was applied to the dataset of 50 AD patients selected from the ADNI database. The SUVR mean of each subject was generated from the optimal final brain region, and age correction for SUVR mean was then carried out. Before and after the age correction, the partial correlation analysis was applied between the SUVR mean and the 3 clinical results. As is evident from Figure 8 and Table 6, the *p* value and *R* coefficient were both significantly improved after age correction. The partial correlation coefficients *R* rose from 0.371 to 0.497 for MMSE, −0.422 to −0.512 for CDRSB, and 0.426 to 0.573 for FDG. During the partial analyses, the effect of education, sex, and ApoE4 status was removed as a set of controlled variables. The results showed that the correlations between the SUVR means values and clinical tests were improved after age-correction. Radiologists may find it easier to diagnose AD by analyzing FDG PET images after age-correction.

As shown in Table 6, we can see that, in subgroups 69–73 and 73–78, the relationship between SUVR mean and two of the clinical results (MMSE and CDRSB for subgroup 69–73 and CDRSB and FDG for 73–78) became much stronger after age correction. Apart from that, the relationship between SUVR mean and FDG in subgroup 56–69 also became much stronger after age correction.

Taken together, it can be safely concluded, with the results of Figure 2 and Tables 3 and 6, that the effect of normal aging becomes apparent after approximately age 50 (Figure 2 and Table 3). Meanwhile, the age of early-onset dementia such as AD has been reported to be 60–65 years [11, 23]. As seen from Table 6, the effect of age correction on subgroup 69–73 and 73–78 was obvious since the *p* values decreases evidently after age correction. We can conclude that the age correction is more effective for ages 69–78.

To verify whether age correction can help to improve the diagnostic accuracy, classification for AD combining PCA and SVM was applied to 50 healthy and 50 AD subjects. Age correction was conducted on the final brain region of IC3 and then the whole brain of each subject was used as the input for the feature extraction of PCA. Leave-one-out cross validation was used during SVM classification to achieve more reliable results by reducing the influence of stochastic factors. As shown in Table 7, the classification performance was achieved with an accuracy of 79% before age correction and 90% after age correction. Table 7 also shows the ROC curves of the two classifications. The AUC values were 0.735 before age correction and 0.84 after age correction.

## 4. Discussion

In the present study, a data-driven analysis of brain glucose metabolism variation across 255 healthy participants revealed a component showing a monotone decrease with an increase in age. According to Figure 4, the regions corresponding to the component IC3 included left inferior and superior frontal gyrus (AAL 15 and AAL 16), the left medial part of superior frontal gyrus (AAL 23), the left medial orbital part of superior frontal gyrus (AAL 25), the right insula (AAL 30), the left anterior cingulate (AAL 31), the left median cingulate, and paracingulate gyri (AAL 33), and bilateral superior temporal gyri (AAL 83 and AAL 84). These findings were mostly in accordance with the typical results that have been reported in most lifespan studies [15–22]. In addition, a set of brain region called the resting state, or default mode network (DMN) of the human brain, which has been suggested by studies of functional connectivity to be relevant to cognitive development, was reported to be significantly related to age [23–25]. This network includes the posterior cingulum (PCC), the precuneus, the left orbital and medial prefrontal cortex (OPFC and MPFC_L), the left orbitofrontal cortex (OFC_L), and the angular gyrus (ANG). As can be observed, the final brain regions overlapped with regions of DMN typically within the left MPFC, OPFC, and medial temporal lobe, which again is in good agreement with the age-related findings of most previous studies described above.

In addition to DMN, the final brain region also covered other areas that have been reported to be related to AD. FDG PET is said to have a predictive value for neurodegeneration at a very early stage [4, 12, 13]. A network was reported to enable FDG PET to detect abnormal metabolism reduction both earlier and at more advanced AD stages than MRI [14]. This network encompassed hippocampal, temporal, parietal, occipital, and posterior caudate regions. It was mostly covered by the final brain regions identified in the present study, with the exception of the occipital regions and right hippocampal regions. Hence, age correction may be conducive to enhancing the conviction of predictions.

**Figure 9:**
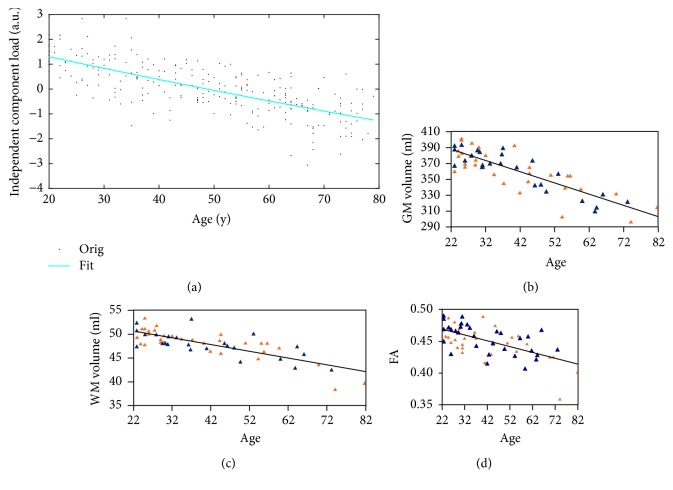
Similar linear scatter plots of negative correlations between age and different imaging indices. (a) Results from the present study. Correlation between SUVR mean and age. Correlations between (a) IC value (b; c; d) Results in [7]. Correlations between (b) GM volume, (c) WM volume, and (d) FA and age.

The IC value of IC3 was found to have a negative linear correlation with age (Figure 2 and Table 3). This finding is in good agreement with reports in the literatures of most regions using FDG PET, where there are clear negative correlations with age [15–22]. In addition, comparison of ICs with the literature [7] showed a similar negative correlation trend through other imaging modalities in GM volume, WM volume, and FA between ages 22 and 82, indicating advanced age-related adverse changes in the brain reflected by various imaging modalities. The results of the comparison between the present study and the literature are shown in Figure 9, suggesting that the definition of IC3 in the present study is credible.

The age-correction model was also conducted on 50 AD subjects. Before the correction, a statistically significant positive correlation between SUVR mean and MMSE or FDG and the negative correlation between SUVR mean and CDRSB were in good accordance with the authors' general understanding of the clinical physiological indices (i.e., the severity of AD is indicated by a lower SUVR mean, a lower MMSE score, and a lower FDG, but a higher CDRSB score). After the correction, these correlations remained unchanged, and the correlation coefficient *R* finally rose to 0.497 for SUVR mean and MMSE, −0.512 for SUVR mean and CDRSB, and 0.573 for SUVR mean and FDG, respectively. The corrected results were compared with those from other studies. In the work by Hatashita [37], the correlation coefficients *R* reached 0.46 for SUVR and MMSE and −0.44 for SUVR mean and CDRSB, which is numerically similar to the correlations before age correction in the present study (0.371 and −0.422). After the age correction, however, the correlation coefficients in the present study rose to 0.497 and −0.512, respectively, indicating a relatively stronger correlation between SUVR and the clinical results. Note that the stronger correlation between SUVR mean and FDG after age correction indicated both potential age- and AD-related responses with areas of angular, temporal, and posterior cingulate indications, which again overlapped with the network of DMN [23–25, 38, 39]. Overall, it can be reasonably concluded that the age correction is valid in clinical AD diagnoses.

The methodological approach used here had two advantages that were crucial in confirming this monotone decreasing component IC3 that revealed a natural decline with age. First, no spatial, age-related, or any other prior information was imposed on the data. Second, the presumption that the components were non-Gaussian independently distributed sources allowed more subtle modes to be detected with respect to abnormal variation and coordinately dominated by certain underlying common features across all subjects. While explaining only a modest amount of the metabolism variation across all 255 healthy subjects (3.7%), the IC3 revealed a strong negative relationship with age (as age explained 51.8% of the spatial variance). Therefore, the FLICA method is very suitable for the present study. In the work by [32], the authors obtained 70 ICs, including IC1, that had an extraordinary relationship with age, for which the *R* square value was 0.9 across 484 healthy participants along with 3 modals (MRI, vertexwise cortical thickness and surface area). In addition, the authors mainly discussed a symmetrical inverted-U IC4 with an *R* square value with age that was 0.5, indicating a mirror effect in age-related brain development. Earlier, in the work by Groves et al. [33], 100 ICs were obtained with a larger dataset that included the same 484 subjects and the three modalities mentioned above, in addition to another three modalities: FA, MD, and MO, all of which were derived from DTI scans. It was understandable that the IC3 obtained during the present study had a lower *R* squared value due to a relatively smaller and single-modal data size, but its relationship with age was still significant.

**Figure 10:**
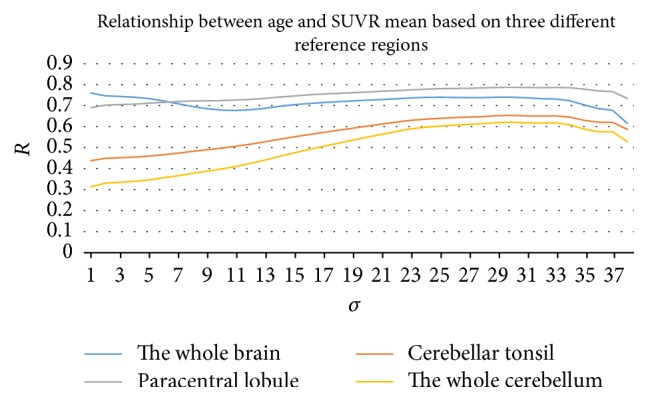
Relationship between age and SUVR mean based on four different reference regions: the cerebellum, the cerebellar tonsil, the paracentral lobule, and the whole brain region.

In addition, the paracentral lobule (AAL 69 and AAL 70) was set during the present study as the reference region when forming SUVR following the research by Zhang et al. [35]. However, other regions may also be selected as reference regions. In the work by Gardener et al. [40, 41], the whole cerebellar or the whole brain region was used as the reference region, and the cerebellar tonsil (AAL 105 and AAL 106) was also reported by Zhang et al. [35] to be one of the two best regions to be a reference, due to their improvement in the separation of natural age-associated changes from changes in brain metabolism. In the present study, these four regions were taken separately as a reference region and the SUVR mean was calculated under different values of *σ*. As shown in Figure 10 (all the results in Figure 10 are the average of the 3000 times Pearson correlation between SUVR mean and age), the relationship between age and SUVR mean was strongest when normalized to the paracentral lobule, with the *R* value climbing up to 0.787 at *σ* = 30. However, its advantage was not obvious when compared with the whole cerebellum (*R* = 0.76 at *σ* = 1). This may be due to the limitations inherent to the datasets, such as possible cohort effects and selection bias, which may influence the use of this more rational reference region to some degree. Nevertheless, it could arguably be concluded that the paracentral lobule can be chosen as the reference region when the age influence needs to be discounted.

## 5. Limitations and Further Considerations

Although certain initial findings regarding brain regions related to age in FDG PET have been explored in the present study, some limitations still exist. First, no cognitive tests such as MMSE or CDRSB have been conducted on subjects at the Huashan Hospital to evaluate their cognitive condition. Further, no partial volume correction (PVC) was performed during the present study due to the lack of MRI data. Both issues should be addressed in future work. Second, the *R* squared value of IC3 was only 0.518, which is far less than for the similar monotonically decreasing IC obtained by [32] whose *R* squared value fitting with age was 0.9. The reason for this discrepancy was likely the limitation of the amount of the subjects' image data, since they had a dataset of 484 subjects. Thus, we could only define one age-correction mathematical model during the long age-span of 20–80 years. Without the data deficiency, we would be able to obtain one age-correction model for each age grade, and the effect of the age correction would then be much more precise. Third, only a linear relationship between the IC value and age was taken into account for the definition of the age-correction mathematical model, since IC3 revealed a monotone linear decrease with increasing age via assessment with a quadratic fit (Figure 2 and Table 3). In fact, a nonlinear relationship with age has also been reported in previous literatures as was found in the present study (e.g., IC11). However, a nonlinear age-correction mathematical model was not investigated but will be studied in the near future. Fourth, in the present study, we used the same template from SPM during the image preprocessing steps for both datasets from Huashan Hospital and the ADNI. However, structural and functional differences between brains in the Chinese and Western elderly populations exist, which may result in various impacts of age on FDG PET scans. Thus, geographical differences between the two datasets from Huashan Hospital and the ADNI will be investigated in the future using different templates. Fifth, in the present study, only FDG PET scans were used for the FLICA. In fact, the FLICA approach can also be used on multimodal data. In addition to FDG PET, the others modals (i.e., other brain imaging techniques) can be MRI, PiB-PET, DTI, and the advanced morphological patterns extracted from these modals, such as FA, MD, and MO from DTI [33], or vertexwise cortical thickness and surface area measures from MRI [32]. Different brain imaging techniques focus on different dementia disorders such as MRI and structural atrophy, PiB-PET and amyloid deposition, and DTI and communication amongst nerve cells in the brain. Thus, combined multimodal analyses can definitely help to increase the number of ICs and obtain more accurate appraisals of brain regions as a result of more integrated information. It is certainly worth comparing the brain regions related to age in these different modals so as to build a corresponding correction template. This approach can also help in the study of differences in how diseases affect each modal of the images. Last of all, the differences in natural brain decline between male and female subjects need to be studied further.

## 6. Conclusion

In summary, the findings of the present investigation suggest that the inferior frontal gyrus, the left medial part of superior frontal gyrus, the left medial orbital part of superior frontal gyrus, the right insula, the left anterior cingulate, the left median cingulate, and paracingulate gyri, and bilateral superior temporal gyri have a strong negative relationship with age in ^18^F-FDG PET images. An age-correction model characterizing the rate of decline of the index SUVR mean provides the possibility of correcting analyses for the effect of the confounding variable, age. By applying the age correction to AD subjects, it was determined that the correction could effectively suppress the interference of age on the analysis and bring disease abnormalities nearer to manifestation. Thus, the method can be applied to patients prior to a clinical AD diagnosis, which will help in determining the severity of the underlying disease.

## Figures and Tables

**Table 1:** Demographic data and cognitive performance of the datasets.

Parameter	Healthy (*n* = 255)	ADNI healthy (*n* = 262)	ADNI AD (*n* = 50)
Demographic data			
Age (years)	49.2 ± 16.7	72.9 ± 4.5	73.1 ± 5.6
Age grades			
20–30	44	-	-
31–40	44	-	-
41–50	42	-	-
51–60	45	3	3
61–70	50	80	10
71–80	30	179	37
Sex (M/F)	128/127	131/131	25/25
EDU	-	16.3 ± 2.7	15.1 ± 3.0
ApoE4	-	0.3 ± 0.5	1.0 ± 0.7
Clinical scales			
MMSE	-	29.0 ± 1.2	23.2 ± 2.0
CDRSB	-	0.0 ± 0.2	4.1 ± 1.3
FDG-PET			
FDG	-	1.3 ± 0.1	1.1 ± 0.1

EDU: education; MMSE: minimental state exam; CDRSB: clinical dementia rating sum of boxes; FDG: average FDG-PET of angular, temporal, and posterior cingulate.

**Table 2:** Information obtained from an IC output by FLICA.

Parameters	Meaning	Utility in this study
IC value	IC value: representing the weight of each subject's intensity explained by the corresponding IC	Used to find out the IC that has the strongest negative relationship with age (see results in Section 3.1.1)
SI	Spatial information: representing the regions that correspond to each IC (Figure 1)	Used to define initial brain regions
VV	Voxel value: the probability operation results of each voxel within the regions of IC (Figure 1)	-
*σ*	The thresholds of VV	Used to determine the more accurate age-related regions (see details in Section 2.4.2)

**Table 3:** Statistical results (quadratic fits) between IC3 and age in the 6 age groups of the 255 subjects.

	Subgroup				Cumulation		
	Number	*p*	*R*		Number	*p*	*R*
20–30	44	0.077	0.117	20–30	44	0.077	0.117
31–40	44	0.672	0.019	20–40	88	2.49**E** − 04	0.177
41–50	42	0.317	0.057	20–50	130	1.11**E** − 09	0.277
51–60	45	**0.032**	0.151	20–60	175	4.37**E** − 18	0.372
61–70	50	**0.01**	0.179	20–70	225	1.56**E** − 31	0.472
71–80	30	0.71	0.026	20–80	255	1.22**E** − 40	0.518

**Table 4:** Statistical results of Pearson correlation analyses between SUVR mean and age under different *σ*.

*σ*	SUVR mean					
	*R*		*p* value		*β*_*C*_	
	Average	±	Average	±	Average	±
1	−0.69078	0.004314	2.15*E* − 36	4.63*E* − 36	−0.00201	1.95*E* − 05
2	−0.70248	0.004163	4.04*E* − 38	9.70*E* − 38	−0.00211	1.99*E* − 05
3	−0.70505	0.004088	1.72*E* − 38	3.48*E* − 38	−0.00213	1.93*E* − 05
4	−0.70732	0.003972	7.79*E* − 39	1.28*E* − 38	−0.00215	1.96*E* − 05
5	−0.71126	0.004001	1.98*E* − 39	3.59*E* − 39	−0.00219	2.00*E* − 05
6	−0.71589	0.003869	3.74*E* − 40	7.52*E* − 40	−0.00225	2.01*E* − 05
7	−0.71954	0.003853	9.62*E* − 41	2.11*E* − 40	−0.00231	2.07*E* − 05
8	−0.72191	0.004004	3.85*E* − 41	9.00*E* − 41	−0.00237	2.18*E* − 05
9	−0.72329	0.00385	2.37*E* − 41	3.97*E* − 41	−0.00243	2.16*E* − 05
10	−0.72439	0.003884	1.62*E* − 41	3.12*E* − 41	−0.00248	2.18*E* − 05
11	−0.72653	0.003878	6.94*E* − 42	1.41*E* − 41	−0.00254	2.28*E* − 05
12	−0.72982	0.003748	2.02*E* − 42	3.85*E* − 42	−0.00261	2.24*E* − 05
13	−0.73463	0.003733	2.96*E* − 43	5.12*E* − 43	−0.00268	2.30*E* − 05
14	−0.74075	0.003678	2.45*E* − 44	4.79*E* − 44	−0.00277	2.36*E* − 05
15	−0.74601	0.003559	3.03*E* − 45	7.36*E* − 45	−0.00285	2.35*E* − 05
16	−0.75093	0.00336	3.60*E* − 46	7.28*E* − 46	−0.00294	2.34*E* − 05
17	−0.75519	0.003342	5.89*E* − 47	1.09*E* − 46	−0.00303	2.37*E* − 05
18	−0.75828	0.003328	1.47*E* − 47	2.85*E* − 47	−0.00311	2.48*E* − 05
19	−0.76163	0.003282	3.26*E* − 48	8.33*E* − 48	−0.0032	2.52*E* − 05
20	−0.76479	0.003203	7.59*E* − 49	1.84*E* − 48	−0.00329	2.51*E* − 05
21	−0.76782	0.00321	1.84*E* − 49	4.31*E* − 49	−0.00338	2.64*E* − 05
22	−0.77095	0.003207	4.29*E* − 50	8.36*E* − 50	−0.00348	2.67*E* − 05
23	−0.77505	0.003002	6.17*E* − 51	1.09*E* − 50	−0.00359	2.69*E* − 05
24	−0.77783	0.003031	1.62*E* − 51	3.07*E* − 51	−0.00368	2.79*E* − 05
25	−0.78031	0.00297	4.91*E* − 52	1.11*E* − 51	−0.00377	2.81*E* − 05
26	−0.78164	0.002894	2.62*E* − 52	5.01*E* − 52	−0.00384	2.84*E* − 05
27	−0.78308	0.00284	1.16*E* − 52	2.12*E* − 52	−0.00392	2.90*E* − 05
28	−0.78417	0.002848	6.79*E* − 53	1.70*E* − 52	−0.00399	2.93*E* − 05
29	−0.78584	0.002811	2.76*E* − 53	6.28*E* − 53	−0.00408	3.05*E* − 05
30	−0.78679	**0.002817**	1.77**E** − 53	3.22**E** − 53	−0.00415	3.05**E** − 05
31	−0.7865	0.002822	2.18*E* − 53	4.26*E* − 53	−0.0042	3.16*E* − 05
32	−0.78558	0.002849	3.41*E* − 53	6.38*E* − 53	−0.00425	3.19*E* − 05
33	−0.78627	0.002835	2.26*E* − 53	4.77*E* − 53	−0.00433	3.28*E* − 05
34	−0.78522	0.002819	3.93*E* − 53	9.98*E* − 53	−0.00439	3.38*E* − 05
35	−0.77836	0.002867	1.15*E* − 51	2.35*E* − 51	−0.00441	3.41*E* − 05
36	−0.77042	0.002963	5.30*E* − 50	1.00*E* − 49	−0.00443	3.51*E* − 05
37	−0.76594	0.00296	4.43*E* − 49	8.09*E* − 49	−0.00453	3.59*E* − 05
38	−0.7343	0.003578	3.35*E* − 43	7.69*E* − 43	−0.00443	4.06*E* − 05

*Note.* All the results in Table 5 are the average of the 3000 Pearson correlations between SUVR mean and age.

**Table 5:** AAL regions covered by the final brain region with *σ* = 30.

Region number	Region name	MNI coordinates (mm)		
		*x*	*y*	*z*
15	Frontal_Inf_Orb_L	−35.98	30.71	−12.11
16	Frontal_Inf_Orb_R	41.22	32.23	−11.91
23	Frontal_Sup_Medial_L	−8.06	15.05	−11.46
25	Frontal_Med_Orb_L	−5.17	54.06	−7.40
30	Insula_R	39.02	6.25	2.08
31	Cingulum_Ant_L	−4.04	35.40	13.95
33	Cingulum_Mid_L	−5.48	−14.92	41.57
83	Temporal_Pole_Sup_L	−39.88	15.14	−20.18
84	Temporal_Pole_Sup_R	48.25	14.75	−16.86

**Table 6:** Statistical results of the age correction in each age group of the 50 AD subjects.

Age grade	Number	Before correction				After correction			
			MMSE	CDRSB	FDG		MMSE	CDRSB	FDG
56–69	11	*p*	0.419	0.166	0.025	*p*	0.158	0.139	0.003
		*R*	0.272	−0.449	0.668	*R*	0.456	−0.476	0.805
69–73	10	*p*	0.037	0.04	0.26	*p*	0.025	0.029	0.251
		*R*	0.663	−0.655	0.394	*R*	0.699	−0.685	0.401
73–78	16	*p*	0.12	0.034	0.011	*p*	0.091	0.02	0.005
		*R*	0.404	−0.531	0.615	*R*	0.436	−0.575	0.664
78–80	13	*p*	0.014	0.067	0.057	*p*	0.015	0.058	0.069
		*R*	0.663	−0.522	0.539	*R*	0.658	−0.537	0.52

*Note. *Age groups 69–71 and 71–73 and 73–76 and 76–78 were combined into one group, respectively, because of their insufficient quantity.

**Table 7:** Classification accuracy for AD before and after age correction.

	Before age correction	After age correction
Accuracy	79%	90%
ROC curves